# New Empirical Evidence on the Validity and the Reliability of the Early Life Stress Questionnaire in a Polish Sample

**DOI:** 10.3389/fpsyg.2017.00365

**Published:** 2017-03-13

**Authors:** Andrzej Sokołowski, Wojciech Ł. Dragan

**Affiliations:** The Interdisciplinary Centre for Behavioural Genetics Research, Faculty of Psychology, University of WarsawWarsaw, Poland

**Keywords:** scale validity, stress, early adversity, psychopathology, ELSQ

## Abstract

**Background:** The Early Life Stress Questionnaire (ELSQ) is widely used to estimate the prevalence of negative events during childhood, including emotional, physical, verbal, sexual abuse, negligence, severe conflicts, separation, parental divorce, substance abuse, poverty, and so forth.

**Objective:** This study presents the psychometric properties of the Polish adaptation of the ELSQ. It also verifies if early life stress (ELS) is a good predictor of psychopathology symptoms during adulthood.

**Materials and Methods:** We analyzed data from two samples. Sample 1 was selected by random quota method from across the country and included 609 participants aged 18-50 years, 306 women (50.2%) and 303 men (49.8%). Sample 2 contained 503 young adults (253 women and 250 men) aged 18–25. Confirmatory and exploratory factor analyses were used to measure ELSQ internal consistency. The validity was based on the relation to psychopathological symptoms and substance misuse.

**Results:** Results showed good internal consistency and validity. Exploratory factor analysis indicates a six-factor structure of the ELSQ. ELS was related to psychopathology in adulthood, including depressive, sociophobic, vegetative as well as pain symptoms. ELSQ score correlated also with alcohol use, but not nicotine dependence. Moreover, ELS was correlated with stress in adulthood.

**Conclusion:** The findings indicate that the Polish version of the ELSQ is a valid and reliable instrument for assessing ELS in the Polish population and may be applied in both clinical and community samples.

## Introduction

Early life stress (ELS) is one of the strongest predictors of adult psychopathology. The Stress Sensitization Model (Hammen et al., 2000) claims that people exposed to ELS are more likely to develop psychiatric disorders. Continuous or frequent stress experiences may lead to increased sensitivity and reactivity to even minor future stressors (Espejo et al., 2007). ELS has, so far, been linked to many disorders, such as depression (Hammen, 2005), anxiety (Espejo et al., 2007), psychotic disorders (Carr et al., 2013), and increased risk of suicide (Dube et al., 2001). Moreover, ELS increases the risk of alcohol abuse (Pilowsky et al., 2009) and smoking (Anda et al., 1999).

Stress contributes to environmental factors that harm, threaten, exceed, or challenge psychological or psychical abilities. These factors include both environmental changes and continuous conditions (Cohen et al., 1995). ELS is related to exposure to an array of stressors during childhood - including emotional, physical, verbal, sexual abuse, death, negligence, severe conflicts, separation, parental divorce, substance abuse, poverty, and so forth (McLaughlin et al., 2010; Pechtel and Pizzagalli, 2011; Baker et al., 2013). Those negative events may lead to prolonged stress, the exhaustion of coping resources, and may impact the hypothalamic–pituitary–adrenal (HPA) axis (Pechtel and Pizzagalli, 2011). The HPA axis is considered to be a neurobiological response system to stress and a crucial element in sustaining stability, as well as physical and mental health (McEwen, 2004).

Due to the importance of early experiences to mental health throughout life, researchers aim to develop reliable tools to estimate the extent of early stress. There are several measurement instruments which assess ELS. The most common are self-reported retrospective questionnaires for adults. These usually cover abuse, negligence, trauma, and other negative events during childhood (Hardt and Rutter, 2004).

The Early Life Stress Questionnaire (ELSQ) is a self-report survey based on the Child Abuse and Trauma Scale, which was reported to have high reliability, validity, and internal consistency (Sanders and Becker-Lausen, 1995). Responders state if they experienced any of 19 negative events during childhood. Items take into account many issues, from physical and sexual abuse to severe domestic conflicts and family separation or social rejection (Cohen et al., 2006b). The ELSQ is widely used in international cohorts with large age ranges (McFarlane et al., 2005). The original version of the ELSQ has a six-factor structure. Factor 1 consists of items involving different types of abuses, domestic violence and negligence. The second deals with breakup of families. Factor 3 deals with family illness. The fourth involves experiences of natural disaster or war. Factor 5 includes illness or hospitalization. The last factor consists of one item referring to being bullied (Cohen et al., 2006b). There is lack of other studies concerning internal structure of the ELSQ, in particular, comparing the factorial structure between distinct cultures.

The ELSQ score has been linked to psychopathology in both clinical and non-clinical samples (McLaughlin et al., 2010; Chu et al., 2013). Cohen et al. (2006b) showed a relation between current anxiety and factors 1, 2, and 6. They also confirmed a correlation between depression and factors 1 and 6. Chu et al. (2013) indicated that interpersonal violation and personal health trauma are particularly related to symptoms of depression and anxiety. These symptoms may also be associated with the time of occurrence of the stressful events (Baker et al., 2013).

A longitudinal study provided evidence that ELS is linked to psychopathology throughout life, including early adulthood and mid-life. Moreover, ELS may not only be associated with the onset of symptoms, but may also be a predictor of persistent psychopathology (Clark et al., 2010). The association between ELS and a wide range of disorders is reported to be prevalent in many countries and cultures (Kessler et al., 2010).

Early life stress is also associated with substance abuse. Those who have experienced high levels of stress prior to puberty are more likely to develop alcohol addiction (Pilowsky et al., 2009) and nicotine dependence in adulthood (Xie et al., 2012). One possible explanation is that substance abuse may be seen as a means of alleviating negative affect and of coping with stress (Nichols and Harlow, 2004). More severe stress is associated with increased risk of early alcohol use (Pilowsky et al., 2009). ELS is associated with starting smoking earlier. It shows a cumulative effect of numerous categories of negative events (Nichols and Harlow, 2004). However, McFarlane et al. (2005) reported that ELSQ was related to nicotine dependency independent of type of stressor, but not to alcohol consumption.

Early Life Stress Questionnaire is commonly used in studies considering the gene-environment interaction as well as brain functional and structural alternations (Cohen et al., 2006a; Saleh et al., 2017). Despite the fact that ELS is commonly studied across clinical subgroups, less is known about the relation between stressful events and the prevalence of disorder indicators in community samples. ELSQ is superior in comparison with other tools for assessment of ELS mainly due to its shortness as well as covering wide range of different childhood adversities. Although the ELSQ is widely used by psychologists, little is known about its psychometric characteristics. Therefore, this study aims to assess the psychometric properties and validity of the Polish adaptation of the ELSQ. We presumed that ELSQ as a measure of ELS will have good internal consistency and factor structure. There is no other early life stress measure validated in Polish, so we used proxy measures (psychopathology symptoms, substance use) to assess the criterion validity. To assess the validity of Polish version of ELSQ in terms of relation to psychopathology we expected that ELSQ score would be a good predictor of psychopathology symptoms, alcohol and nicotine use. We hypothesized that (a) ELSQ has a six-factor structure as was reported in its original version (Cohen et al., 2006b), (b) ELSQ score will correlate positively with the Symptom Checklist-27-plus score (SCL-27-plus; Hardt, 2008), (c) ELSQ score will correlate positively with the Alcohol Use Disorders Identification Test score (AUDIT; Saunders et al., 1993), (d) ELSQ score will correlate positively with the Fagerström Test for Nicotine Dependence score (FTND; Heatherton et al., 1991), (e) there will be positive correlation between ELSQ score and Recent Life Changes Questionnaire score (RLCQ; Rahe, 1975).

## Materials and Methods

### Participants and Procedure

Two samples took part in our study. Sample 1 consisted of a total number of 611 adults from community sample. Two participants were excluded, as they did not answer all questions, leaving 609 respondents (306 women and 303 men) aged 18–50 (*M* = 34.33; *SD* = 8.91). Levels of education ranged from 2 to 24 years. Respondents were selected from across the whole country by random quota, i.e., random selection from stratified sampling. They came from various socioeconomic and educational backgrounds.

Sample 2 took part in the study to confirm the internal structure of ELSQ as well as to test the relation between ELS and stressors in adulthood. 503 young adults (253 women and 250 men) aged 18–25 (*M* = 21.41; *SD* = 1.88) were recruited from Warsaw and surrounding area. Level of education ranges from 1 to 22 years.

Participants from sample 1 completed the ELSQ, SCL-27-plus, AUDIT, and FTND. Due to the sensitive nature of ELSQ questions, the self-reporting procedure was used. Data from the FTND were collected from 197 smokers. The Scale of Material Remuneration was used as a socioeconomic status (SES) indicator and was calculated for 469 participants (excluding students, pensioners and the unemployed). Exploratory factor analysis (EFA), correlations between ELS and psychopathology symptoms, alcohol and nicotine usage, as well as regression analyses were carried out in this sample.

Sample 2 completed the ELSQ and RLCQ. Confirmatory factor analysis (CFA) and correlation between ELS and recent life changes were carried out in this sample. **Table 1** shows descriptive statistics for both samples.

**Table 1:** Descriptive statistics.

	*Min*	*Max*	*M*	*SD*
**Sample 1**				
Age	18	50	34.33	8.91
Education	2	24	12.47	3.04
SES	18.20	66.20	24.26	5.76
FTND	0	10	4.48	2.10
AUDIT	0	29	4.18	5.40
SCL27+ GI	25	83	37.00	11.72
SCL27+ SP	5	20	6.80	2.70
SCL27+ VG	5	20	7.69	2.74
SCL27+ PN	6	22	10.30	3.62
SCL27+ AG	4	16	4.99	2.02
SCL27+ DC	5	20	6.58	2.63
SCL27+ DL	0	5	0.64	1.27
ELSQ	0	10	1.28	1.90
**Sample 2**				
Age	18	25	21.41	1.88
Education	1	22	13.21	2.71
ELSQ	0	13	2.97	2.93
RLCQ	0	42	11.10	7.28


*AUDIT, Alcohol Use Disorders Identification Test; ELSQ, Early Life Stress Questionnaire; FTND, Fagerström Test for Nicotine Dependence; SES, socioeconomic status; SCL27+ AG, The Symptom Checklist-27-plus Agoraphobic symptoms; SCL27+ DC, The Symptom Checklist-27-plus Depressive Current symptoms; SCL27+ DL, The Symptom Checklist-27-plus Depressive Lifetime symptoms; SCL27+ GI, The Symptom Checklist-27-plus Global Index; SCL27+ PN, The Symptom Checklist-27-plus Pain symptoms; SCL27+ SP, The Symptom Checklist-27-plus Sociophobic symptoms; SCL27+ VG, The Symptom Checklist-27-plus Vegetative symptoms.*

There were significant differences between two samples in terms of age, years of education, and ELSQ scores (*p* < 0.001) mainly as a result of different forms of recruiting the participants. Sample 1 included also participants in middle adulthood. Sample 2 was more educated and had higher mean ELSQ score.

This study was carried out in accordance with the recommendations of the local ethic committee. All subjects gave informed consent in accordance with the Declaration of Helsinki.

### Measures

Nineteen items of the ELSQ measure exposure to stressful events in childhood. Questions deal with negligence, abuse, death in family, sustained domestic conflicts, and so forth. All items were translated by the authors and back translated to English by a professional translator. Results were compared to the original questionnaire. No major change was made in the Polish version of the ELSQ. Data on the prevalence of each event in our sample are shown in **Table 2**. The global index score was calculated by summing up the number of adverse events.

**Table 2:** Prevalence of self-reported early life stress (ELS) events.

	Total (%)	Women (%)	Men (%)
Premature birth	40 (6.58)	25 (8.20)	15 (4.95)
Adoption	8 (1.32)	7 (2.30)	1 (0.33)
Surgery/hospitalization	57 (9.42)	26 (8.55)	31 (10.30)
Major illness (self)	39 (6.43)	15 (4.93)	24 (7.92)
Bullied/social rejection	16 (2.65)	8 (2.65)	8 (2.65)
Physical abuse	44 (7.30)	15 (4.98)	29 (9.60)
Sexual abuse	4 (0.66)	4 (1.33)	0 (0)
Emotional abuse	66 (10.96)	32 (10.63)	34 (11.30)
Poverty/neglect	21 (3.50)	9 (3.00)	12 (4.00)
Natural disaster	72 (11.88)	27 (8.89)	45 (14.90)
Destroyed home	9 (1.48)	3 (0.99)	6 (1.98)
War	3 (0.49)	2 (0.66)	1 (0.33)
Divorce	60 (9.97)	32 (10.60)	28 (9.33)
Separation from family	16 (2.65)	6 (1.99)	10 (3.31)
Severe family conflict	62 (10.28)	28 (9.27)	34 (11.30)
Death: parent/sibling	131 (21.65)	72 (23.76)	59 (19.54)
Major illness in family	84 (13.93)	47 (15.56)	37 (12.29)
Domestic violence	51 (8.49)	21 (6.98)	30 (10.00)
Other	40 (6.68)	20 (6.69)	20 (6.67)


The SCL-27-plus was used as a multidimensional screening tool for mental health problems. As well as a global severity index it provides subscales of depressive, sociophobic, vegetative, pain, and agoraphobic symptoms. Each scale is composed of four to six items. It is based on the full version of SCL-90-R. Both versions have similar high internal consistency as well as sensitivity and specificity, with the short version showing even better psychometric properties (Kuncewicz et al., 2014).

We used the FTND (Heatherton et al., 1991) to assess nicotine dependence. It has satisfactory reliability and consists of 6 questions, such as time to first cigarette of the day and number of cigarettes per day. The maximum score is 10. Reliability was assessed by estimating internal consistency using Cronbach’s α, which was α = 0.62 in our sample.

The AUDIT (Saunders et al., 1993) is a widely used self-report measure of abnormal drinking behavior and problems related to hazardous or harmful substance use. The questionnaire contains 10 items scored from zero to four with a maximum score of 40. The response scale is based on frequency, i.e., recurrent intoxication, and ranges from 0 (“never”) to 4 (“daily”). Cronbach’s α in our sample was 0.89.

The RLCQ (Rahe, 1975) was used to assess the stress in adulthood. It consists of 55 items that reflects the impact of recent stressful events. Participants reported if they experienced any of them in the previous 24 months. RLCQ has high reliability (α = 0.99).

The Scale of Material Remuneration was used as a SES indicator. It is based on the average income assigned to individual occupations, covering both occupation and income indices (Domański et al., 2009).

### Data Analysis

To test the sex differences in ELSQ, SCL-27-plus, FTND, and AUDIT in first sample the Student’s *t*-test was used. Cohen’s *d* method was used to estimate the effect size of sex differences and *d*s of 0.20, 0.50, 0.80 refer to small, medium and large effect sizes, respectively (Cohen, 1988). We used EFA to define the factorial structure of ELSQ in sample 1. CFA in sample 2 was used to confirm structure obtained in previous analysis. To assess the validity of ELSQ we correlated its global index with psychopathology symptoms and substance usage. Moreover, the relation of ELSQ to recent life stress was determined through correlation (Pearson coefficient). Regression analyses were used to determine the ELSQ, sex, age, and SES contribution to psychopathology symptoms, FTND, and AUDIT outcomes. All assumptions of tests were met.

## Results

### Prevalence of ELS

From a total of 609 participants in sample 1, 303 (49.8%) reported having been exposed to at least one ELS. The most frequent were the death of a relative, major illness in the family, natural disasters and emotional abuse. Moreover, 182 (29.9%) respondents reported to have more than one event and 117 (19.2%) at least three. **Table 2** presents the prevalence of each ELS event.

We tested for any sex differences in the measured indices in our sample. Men scored significantly higher on the AUDIT and FTND. Women scored higher on the SCL-27-plus global index and the SCL-27-plus current depression indicator. There was no difference in ELSQ outcome. All statistical indicators are presented in **Table 3**.

**Table 3:** Sex differences.

	Women *M* (*SD*)	Men *M* (*SD*)	*t*	*p*	*r*
FTND	3.80 (2.11)	4.89 (2.00)	–3.655	0.000	0.53
AUDIT	2.56 (4.35)	5.82 (5.85)	–7.803	0.000	0.63
SCL27+ GI	37.94 (11.92)	36.05 (11.45)	–1.997	0.046	0.16
SCL27+ SP	6.97 (2.81)	6.62 (2.58)	–1.634	0.103	
SCL27+ VG	7.83 (2.69)	7.55 (2.78)	–1.287	0.199	
SCL27+ PN	10.54 (3.73)	10.05 (3.50)	–1.671	0.095	
SCL27+ AG	5.08 (2.03)	4.91 (2.00)	–1.025	0.306	
SCL27+ DC	6.83 (2.85)	6.32 (2.37)	–2.418	0.016	0.19
SCL27+ DL	0.68 (1.32)	0.60 (1.22)	–0.769	0.442	
ELSQ	1.21 (1.86)	1.34 (1.94)	–0.850	0.396	


*For abbreviations see **Table 1**.*

### Exploratory Factor Analysis

The factorial structure of Polish version of the ELSQ was evaluated with EFA. We analyzed all 19 items with principal axis factoring followed by direct oblimin rotation. Six factors were extracted, which explain 37% of the total variance. The first six eigenvalues were 3.73, 1.75, 1.66, 1.30, 1.16, and 1.08. Factor loadings are shown in **Table 4**. Factors were intercorrelated (correlation ranged from 0.03 to 0.34).

**Table 4:** Exploratory factor analysis (EFA).

	Factor 1	Factor 2	Factor 3	Factor 4	Factor 5	Factor 6
Physical abuse	0.76					
Emotional abuse	0.55					
Domestic violence	0.47			0.44		
Bullied		0.71				
Adoption		0.62				
Major illness in family			0.62			
Death in family			0.51			
Natural disaster			0.47			
Neglected			0.32			
Severe family conflict				0.64		
Divorce				0.62		
Separated from family				0.49		
Other				0.39		
Major illness (self)					–0.79	
Hospitalization / surgery					–0.48	
Sexual abuse						0.41
Premature birth						0.38
War						
Fire destroyed home						


One item (*domestic violence*) loaded two factors. Two items, *war* and *fire destroyed home*, did not reach the inclusion criterion of 0.32 (0.13 and 0.22, respectively) and did not account for any factor. We assessed reliability by estimating internal consistency using Cronbach’s α, which was α = 0.74 for the ELSQ.

### Confirmatory Factor Analysis

We used CFA to assess the adequacy of the factor structure previously described (see **Table 4**). CFA was performed using AMOS 23. Goodness-of-Fit Index and Comparative Fit Index were 0.94 and 0.87, respectively. Root Mean Square Error of Approximation was 0.06. Therefore, goodness of fit indices were satisfactory and six-factor model fit the data in sample 2.

### Validity

To assess the relation between ELS, psychopathology symptoms and substance usage we correlated ELSQ global index scores with outcomes from other questionnaires. As predicted, ELSQ was significantly related to SCL-27-plus scores, both its global index (*r* = 0.25) and subscales: *sociophobic* (*r* = 0.24), *vegetative* (*r* = 0.22), *pain* (*r* = 0.24), *depressive current* (*r* = 0.16), and *depressive lifetime* (*r* = 0.25), but not agoraphobic symptoms. ELSQ was also correlated with AUDIT (*r* = 0.19), but not with FTND.

The contributions of ELSQ, age, and SES to FTND, AUDIT, and SCL-27-plus outcomes were assessed with regression equations. The sex factor significantly differentiated the results of the FTND, AUDIT, and SCL-27-plus, which were included in regression models. Regression analyses are shown in **Table 5**.

**Table 5:** Regression analyses.

	*β*	*t*
**SCL27+**		
ELSQ	0.25^∗∗^	5.60
Sex	0.08	1.70
Age	0.09	1.90
SES	-0.01	-0.20
**FTND**		
ELSQ	0.07	0.87
Sex	-0.27^∗^	-3.42
Age	0.17^∗^	2.19
SES	-0.13	-1.65
**AUDIT**		
ELSQ	0.21^∗∗^	4.77
Sex	-0.30^∗∗^	-6.94
Age	0.08	1.93
SES	-0.04	-0.99


*^∗^*p* < 0.05; ^∗∗^*p* < 0.001; For abbreviations see **Table 1**.*

Hierarchical multiple regression analysis was performed with ELSQ, sex, age, SES as predictors, entered respectively, and FTND as dependent variable. Sex (β = –0.27; *p* = 0.001) and age (β = 0.17; *p* = 0.03) were significant predictors, but they showed minimal effect size. ELSQ and SES were not significant predictors. The model was well fitted to the data *F*(4,151) = 4.83; *p* = 0.001 and explained 9% of the variance (*R*^2^ = 0.09).

Correspondingly, we conducted regression analysis with ELSQ, sex, age, and SES as predictors and AUDIT as dependent variable. Sex (β = –0.30; *p* = 0.000) was significant and showed a small effect size as a predictor, and ELSQ (β = 0.21; *p* = 0.000) was significant but showed minimal effect size as a predictor of AUDIT. Age and SES were not significant predictors. The model was well fitted to the data *F*(4,464) = 19.92; *p* = 0.000 and explained 13.9% of the variance (*R*^2^ = 0.139).

Analogously, we performed regression analysis with ELSQ, sex, age, and SES as predictors and SCL-27-plus global index as dependent variable. Only ELSQ (β = 0.25; *p* = 0.000) was a significant predictor of SCL-27-plus, having minimal effect size. Sex, age, and SES were not significant predictors. We found a well-fitted model *F*(4,464) = 9.92; *p* = 0.000, which explained 7.1% of the variance (*R*^2^ = 0.071).

Furthermore, we tested the relation between ELS and recent life stress. ELSQ correlated positively with RLCQ (*r* = 0.59).

## Discussion

The psychometric quality of the Polish version of the ELSQ was assessed. We determined that it has satisfactory reliability, validity and internal consistency. The prevalence of adverse events in an international cohort is slightly different than in our sample. The most frequent events measured by original version of the ELSQ were divorce, family conflict, and being bullied. However, the least prevalent stressors (*war*, *sexual abuse*, *adoption*, and *destroyed home*) were the same (see Cohen et al., 2006b). Interestingly, we found a different number of people who reported at least one adverse event than did Cohen et al. (2006b). In that study 72.4% of participants reported at least one adverse event, while in our study only 49.8% did. It should be noted, however, that there are some substantial differences between our study and Cohen et al.’s. There were differences in the method of selection of respondents. Participants in our study were selected by random quota, while Cohen et al.’s study recruited by advertisement. There were also different age ranges of participants. It should be also remembered that Cohen et al.’s sample spanned three western countries, while ours consisted only of Polish citizens.

We replicated the original six-factor structure of the ELSQ. However, our internal structure was different to the initial one. All factors were loaded by at least two items. Particular items are qualitatively distinct and may not contribute to homogenous factors. The item *war* did not load any factor, probably because only three respondents reported its occurrence. It is unlikely that Polish people aged 18–50 have experienced such an event. Moreover, Chu et al. (2013) also did not replicate the original structure in a community sample. They found five components of the ELSQ. *Home destroyed* was also dropped in our study due to minimal prevalence. Considering correlation between factors, the use of the ELSQ global index may be suggested.

Early Life Stress Questionnaire was a significant predictor of AUDIT and SCL-27-plus outcomes, indicating that it may indeed be related to adult psychopathology and the development of alcohol misuse. As hypothesized, ELSQ was related to psychopathological indices, including depressive, sociophobic, vegetative, and pain symptoms. These relations emphasize the high validity of ELSQ. Agoraphobic symptoms were not related to ELS. Brown and Harris (1993) study exhibit that although anxiety is strongly related to ELS, the agoraphobia showed the weakest association, suggesting that distinct adversities may make different contributions (e.g., the parental indifference having stronger impact on agoraphobia than sexual abuse). Moreover, Magee (1999) reported that the strongest predictor of agoraphobia was the occurrence of life threatening accidents, combat in war (for men), and fire/flood or other natural disaster during childhood. As mentioned above, *war* was the least prevalent stressor in our study. Thus, it is possible that the weak relation between ELSQ and agoraphobic symptoms, as measured by SCL-27-plus, is due to the specific prevalence of early stressors in our sample. The strongest relationship we observed was between ELSQ and vegetative and pain symptoms. This relationship is confirmed by the results of many studies showing that ELS is just as significant for physical health as for mental health (Coles et al., 2015; Friborg et al., 2015).

We did not find an impact of ELS on nicotine usage. Many previous studies confirmed the relationship between ELS and smoking (e.g., McFarlane et al., 2005). Smoking, however, may be seen as a way of coping with everyday stress, independent of early life experience, and nicotine usage may also be associated with factors other than stress. Smoking, as a multidimensional, behavior, may not only be associated with regulation of negative affect (due to multiple stressors), but also with an increase of positive affect or become an automatic habit (Hudmon et al., 2003). Moreover, smoking may serve numerous functions (e.g., simplification of social interaction). In general, smokers should be treated as a heterogeneous group. It should be also noted that the relation between ELS and nicotine usage might be mediated or moderated by genetic or personality factors (e.g., Ansell et al., 2012; Mingione et al., 2012), which were not controlled for in our study.

Moreover, we confirmed the relation between ELS and recent life stress. It is in line with stress sensitization theory which underline that experiencing highly traumatic or threatening events may lower the threshold for reactivity and decrease resilience to subsequent stressors in adolescence and adulthood. Thus, ELS may be associated with developing psychopathology in adulthood (Hammen et al., 2000; Benjet et al., 2010).

The present study has limitations. The use of a retrospective instrument may result in recall bias. However, Hardt and Rutter, 2004 showed that such bias is not large enough to invalidate the results of retrospective studies of childhood adversities. The second limitation is the lack of clinical assessment. Nonetheless, we used a screening tool for the symptoms of disorders. Our study’s strong point is that the sample spans the whole country. We also investigated many stressors - not only abuse, but also negligence and social rejection.

The Polish adaptation of the ELSQ is a reliable and valid instrument for estimating ELS. Considering the growing interest in early experience, it may be a useful tool for both clinicians and researchers.

## Author Contributions

All authors contributed and participated in the preparation of the article and all research steps: AS and WD designed this work, coordinated the data collection, performed the analyses, and wrote the manuscript.

## Conflict of Interest Statement

The authors declare that the research was conducted in the absence of any commercial or financial relationships that could be construed as a potential conflict of interest.
